# Changing on the Concentrations of Neonicotinoids in Rice and Drinking Water through Heat Treatment Process

**DOI:** 10.3390/molecules28104194

**Published:** 2023-05-19

**Authors:** Ziyang Wei, Bo Zhang, Xu Li, Yanxia Gao, Yuan He, Jingchuan Xue, Tao Zhang

**Affiliations:** 1 School of Environmental Science and Engineering, Sun Yat-Sen University, Guangzhou 510275, China; 2 Key Laboratory for City Cluster Environmental Safety and Green Development of the Ministry of Education, School of Ecology, Environment and Resources, Guangdong University of Technology, Guangzhou 510006, China

**Keywords:** neonicotinoids, imidacloprid, heat treatment, rice, drinking water

## Abstract

Neonicotinoids (NEOs) have become the most widely used insecticides in the world since the mid-1990s. According to Chinese dietary habits, rice and water are usually heated before being consumed, but the information about the alteration through the heat treatment process is very limited. In this study, NEOs in rice samples were extracted by acetonitrile (ACN) and in tap water, samples were extracted through an HLB cartridge, then, a high-performance liquid chromatography system and a triple quadrupole mass spectrometry (HPLC-MS/MS) were applied for target chemical analysis. The parents of NEOs (p-NEOs) accounted for >99% of the total NEOs mass (∑NEOs) in both uncooked (median: 66.8 ng/g) and cooked (median: 41.4 ng/g) rice samples from Guangdong Province, China, while the metabolites of NEOs (m-NEOs) involved in this study accounted for less than 1%. We aimed to reveal the concentration changes of NEOs through heat treatment process, thus, several groups of rice and water samples from Guangdong were cooked and boiled, respectively. Significant (*p* < 0.05) reductions in acetamiprid, imidacloprid (IMI), thiacloprid, and thiamethoxam (THM) have been observed after the heat treatment of the rice samples. In water samples, the concentrations of THM and dinotefuran decreased significantly (*p* < 0.05) after the heat treatment. These results indicate the degradation of p-NEOs and m-NEOs during the heat treatment process. However, the concentrations of IMI increased significantly in tap water samples (*p* < 0.05) after heat treatment process, which might be caused by the potential IMI precursors in those industrial pesticide products. The concentrations of NEOs in rice and water can be shifted by the heat treatment process, so this process should be considered in relevant human exposure studies.

## 1. Introduction

Neonicotinoids (NEOs) have gradually become the most widely utilized class of insecticides in the world since imidacloprid (IMI) was introduced in the mid-1990s [1]. In early studies, the toxicity of NEOs was considered limited to mammals, including the human race [2]. Furthermore, recent studies revealed some serious effects of NEOs on mammals, including reproductive toxicity [3], neurotoxicity [4], and the sublethal effect [3,4], and some metabolites of NEOs (m-NEOs) have been confirmed to be more toxic than the parents of NEOs (p-NEOs) [4,5]. Humans have been extensively exposed to NEOs. The urinary and serum concentrations of NEOs, especially m-NEOs [6], in human biological monitoring, have been frequently reported in different countries [6,7,8,9,10].

Dietary exposure is one of the most prominent human exposures to NEOs. In a previous study, NEOs were significantly detected in fruits and vegetables, with concentrations up to 101 ng/g, and IMI had the highest concentration [5]. NEOs were also detected in the brown rice, millet, oats, and corn grown in China, and thiamethoxam (THM) and IMI were the most commonly detected NEOs in these grains, with concentrations up to 102 ng/g [11]. Moreover, NEOs were frequently detected in surface water, groundwater, and drinking water from China [12,13,14]. The daily exposure of humans to many chemicals, including NEOs, is very significant [15,16,17]. According to Chinese dietary habits, rice and water are usually cooked or boiled before being consumed. However, information on NEOs in rice and water through the heat treatment process is very limited; only one study reported the reduction in dinotefuran (DIN) in rice through the cooking process [18].

In this study, a regional survey about NEOs in rice and tap water was conducted in Guangdong Province, Southern China. The concentrations of six p-NEOs, i.e., acetamiprid (ACE), IMI, clothianidin (CLO), thiacloprid (THD), THM, and DIN, and three m-NEOs, i.e., N-Desmethyl-acetamiprid (N-dm-ACE), olefin-imidacloprid (olefin-IMI), and 1-methyl-3-(tetrahydro-3-furylmethyl) urea (DIN-U), were determined. Subsequently, the concentration changes of NEOs were obtained before and after the heat treatment process to clarify the possible alteration.

## 2. Results and Discussion

### 2.1. Concentrations of NEOs in Rice and Drinking Water

**Figure 1:**
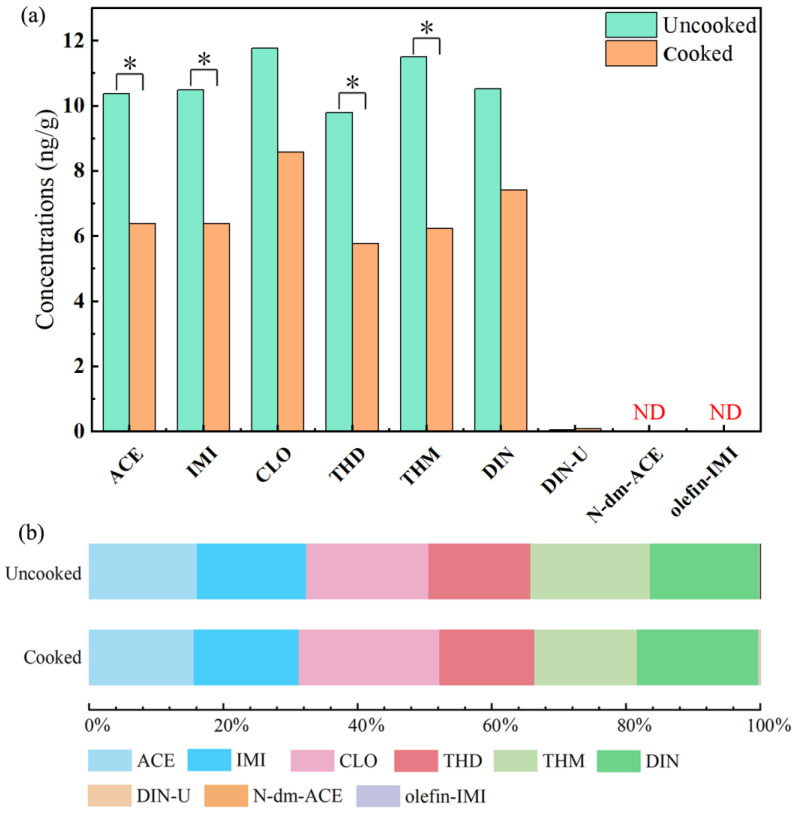
Median concentrations of NEOs in rice before and after the cooking process (plot (**a**)) and the proportions of each individual NEO in ΣNEOs (plot (**b**)). The single asterisk (*) represents a significant difference (*p* < 0.05, *t*-test), and ND means not detected.

The concentrations of NEOs in the paired uncooked and cooked rice samples (n = 35 pairs) collected from Guangzhou City and Qingyuan City, Guangdong Province, China, are shown in Table 1. In the uncooked rice, all six-target p-NEOs were widespread, with the detection rates (DRs) ranging from 69% (DIN) to 91% (ACE), and at least one p-NEO was detected in 97% of the uncooked rice samples. The median concentrations of the six p-NEOs range from 9.79 (THD) to 11.8 (CLO) ng/g (Table 1 and Figure 1), and the proportion of the individual p-NEOs are similar (Figure 1). Three target m-NEOs were also detected in the uncooked rice samples, with the DRs in the range of 46% (N-dm-ACE and olefin-IMI) and 54% (DIN-U), and the median concentrations of the m-NEOs range from 0.01 (olefin-IMI) to 0.06 (DIN-U) ng/g. In the cooked rice, six target p-NEOs were also frequently detected, with DRs ranging from 57% (DIN) to 89% (ACE), and the probability of at least one p-NEO being detected is 97%. The median concentrations of the six p-NEOs range from 5.77 ng/g to 8.58 ng/g (Table 1 and Figure 1) in the cooked rice. CLO (8.58 ng/g) had the highest median concentration, followed by DIN (7.41 ng/g), IMI (6.39 ng/g), ACE (6.38 ng/g), THM (6.24 ng/g), and THD (5.77 ng/g). Three target m-NEOs were detected with DRs of 34%, 40%, and 60% for olefin-IMI, N-dm-ACE, and DIN-U, respectively, and the median concentrations of the m-NEOs in the cooked rice range from 0.01 (olefin-IMI) ng/g to 0.09 (DIN-U) ng/g. In addition, the concentrations of ACE, IMI, CLO, THD, THM, and DIN in tap water from the laboratory were 3.94, 4.10, 3.79, 3.45, 4.82, and 2.46 ng/L, respectively, which were three orders of magnitude lower than those in rice. DIN-U, N-dm-ACE, and olefin-IMI were hardly detected in tap water from the laboratory with concentrations below 0.1 ng/L.

**Figure 2:**
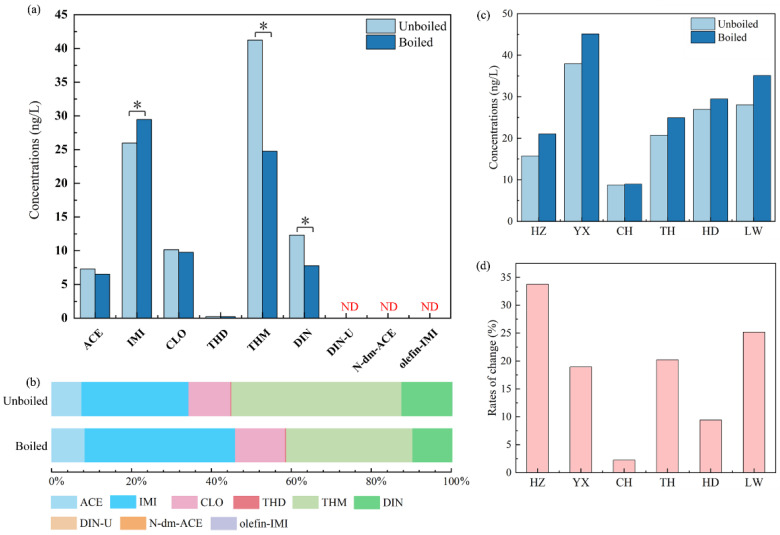
Median concentrations of NEOs in tap water samples obtained from six districts in Guangzhou before and after being boiled (plot (**a**)), the proportions of each individual NEO in ΣNEOs (plot (**b**)), median concentrations of IMI in six sample groups (plot (**c**)), and the median rates of IMI concentration changing (plot (**d**)). The single asterisk (*) represents a significant difference (*p* < 0.05, *t*-test), and ND means not detected. HZ means Haizhu district, YX means Yuexiu district, CH means Conghua district, TH means Tianhe district, HD means Huadu district, and LW means Liwan district.

In the tap water samples collected from Guangzhou, the median concentrations of the six p-NEOs range from 0.23 ng/L to 41.3 ng/L (Figure 2). THM (41.3 ng/L) had the highest median concentration, followed by IMI (26.0 ng/L), DIN (12.3 ng/L), CLO (10.2 ng/L), ACE (7.28 ng/L), and THD (0.23 ng/L), while the median concentrations of the three m-NEOs were lower than the LOQ. In the boiled tap water, IMI (29.5 ng/L) had the highest median concentration among the six p-NEOs, followed by THM (24.7 ng/L), CLO (9.76 ng/L), DIN (7.76 ng/L), ACE (6.50 ng/L), and THD (0.22 ng/L), while the three m-NEOs were still undetected. The p-NEOs concentrations we obtained in the tap water are generally higher than those from a Chinese nationwide baseline survey on NEOs in tap water (median values: 0.38–7.59 ng/L) [14], and the THM concentration in the tap water collected in this study was extremely higher (41.3 ng/L compared with the 4.50 ng/L median concentration observed in the Chinese nationwide baseline survey, indicating an approximately one order of magnitude difference). On the one hand, this result indicates that the p-NEO pollution in the tap water of Guangzhou is more significant than the national average. On the other hand, NEOs generally exhibit more persistence in conventional water treatment than in emerging water treatment [19], and the traditional treatment process cannot efficiently remove NEOs [19,20]. Previous studies reported comparable IMI, THM, DIN, and ACE removal rates in WWTP [20], while another research indicates that conventional drinking water treatment could hardly remove IMI [21]. Emerging treatment processes present outstanding efficiencies in NEO removal. For example, granular activated carbon (GAC) filtration could remove nearly 100% of IMI, THM, and CLO [19], and the removal efficiencies of more NEOs increased when the ozone process was introduced [21]. Thus, our findings on the NEOs in tap water might be a warning signal to reveal the limited NEO removal efficiency of the wastewater treatment plant (WWTP) in Guangzhou and upgrade the water treatment process. Furthermore, the high concentration of THM was probably caused by the temporal and spatial volatilities of tap water, which were observed in a previous study. The maximum median concentration of THM ranges from <0.03 ng/L (in Yunnan Province) to 74.7 ng/L (in Guangxi Province) in China [14].

The results on the composition profiles of NEOs in rice are associated with the production and consumption pattern of NEOs in China [22,23]. In this study, CLO had the highest mean concentration in the rice samples, followed by THM, IMI, and DIN. High profiles of these four NEOs were also observed in the tap water samples. The results could be attributed to the high market shares of THM (36%), IMI (35%), and CLO (14%) in the total NEOs in China [23,24]. Moreover, the usage and emission of DIN in China have been increasing in recent years [23,25,26], and DIN is reportedly one of the main “new generation” NEOs, which has been gradually replacing the “old NEOs” (i.e., IMI and ACE) in China [27]. Furthermore, our previous study extensively detected p-NEOs in the human urine samples collected in China [28], with CLO, IMI, THM, and DIN collectively accounting for 98% of the ∑p-NEOs, which is consistent with the results of the current study. Interestingly, the median concentrations of three m-NEOs were less than 0.10 ng/g and not detected in the rice and tap water samples, but they were frequently detected in human urine samples in China [6]. These findings indicate that rice and drinking water might not be important sources of m-NEOs in people from Guangzhou, and m-NEOs might be metabolized from the p-NEOs in rice and drinking water or ingested from other sources. Several studies have proven that the metabolism of p-NEOs could occur in plants and animals through various pathways, including ring opening, demethylation, nitro-reduction, and cyano-hydrolysis [29,30,31,32]. The in vivo metabolism tendency of p-NEOs might result in increased concentrations of m-NEOs in humans.

The composition profiles of NEOs in tap water are quite different from those in rice (Figure 2), especially the median concentrations of THM and IMI, which are much higher than that of ACE, CLO, and DIN, while THD was hardly detected. The concentration of each NEO is generally 2–3 orders of magnitude lower in the tap water samples than in the rice samples, and the concentration of CLO only ranks fourth among all the p-NEOs in the tap water samples. Moreover, the concentrations of IMI (72.5 ng/L), THM (53.2 ng/L), ACE (34.4 ng/L), and CLO (25.2 ng/L) in the surface water samples collected from Pearl River in Guangzhou [33] are also significantly higher than those in the tap water samples. IMI and THM are the dominant NEOs analytes in the current study (Figure 2), which is also supported by Yi et al. [33]. Moreover, the detection of THD is also comparable to that of other NEOs in the surface water of Guangzhou [33]. A previous study indicates that the NEO concentrations in tap water are lower than those in surface water, and drinking water treatment plants eliminate approximately 50% of NEOs from surface water [34]. However, the removal efficiencies of NEOs in water treatment plants vary due to different treating processes, such as GAC filtration, which could remove nearly 100% of IMI, THM, and CLO [19]. Therefore, the NEO concentrations in the source water might affect the level in the tap water, and a part of the NEOs could be removed through water treatment processes. In addition, the chlorine in tap water could also induce the transformation of a part of the NEOs [19]. The half-lives for CLO and IMI are ~2.5 and ~70 days in tap water within 5 mg/L (as Cl2) chlorine, and THM exhibits no significant loss [19], which might contribute to the lower concentration of CLO in tap water compared with THM and IMI.

### 2.2. Fates of NEOs in Rice through Heat Treatment Process

Our results indicate that four p-NEOs (i.e., ACE, IMI, THD, and THM) decreased significantly (*p* < 0.05, *t*-test), and CLO and DIN also presented a decline (Figure 1) in the rice samples after the cooking process. We further estimated the reduction rates (RRs) of p-NEOs in rice through the cooking process (Equation (1)), while the RRs of the m-NEOs were not calculated due to their extremely low concentrations.

(1) $$\mathrm{RR}=\left(C_{1}-C_{2}\right)/C_{1},$$

where *C*_1_ and *C*_2_ are the median concentrations (ng/g) of each NEO in uncooked and cooked rice, respectively, and the RRs of the p-NEOs range from 27% to 46% in rice. The profiles of the p-NEOs in rice samples before and after the heating process show no change (Figure 1b). Our results are supported by previous studies. Watanabe et al. [18] reported a positive RR value (40%) of DIN in rice after the cooking processes. Significant reductions in several pesticides in rice after the cooking process were also observed in two Japanese studies [35,36]. In addition, Hanafi et al. [37] suggest that ACE is reduced after the heat process in pre-washed okra fruits with a RR of 90%. These observations reveal that the contents of various p-NEOs decreased significantly in the rice samples after the heat process.

Volatilization and chemical transformation were inferred to be the two causes of the reductions. Therefore, steam samples were collected by a distillation unit in another cooked rice sample. According to Table S1, the concentrations of the NEOs in the steam were negligible (ranging from <0.001 ng/L to 0.012 ng/L) compared with those in rice. Therefore, the influence of volatilization on the reduction in NEOs during the heat process could be ignored, and NEOs might experience degradation.

**Figure 3:**
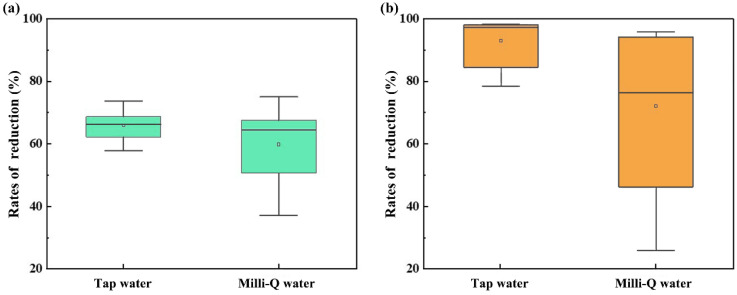
The reduction rates of ∑p-NEOs (plot (**a**)) and ∑m-NEOs (plot (**b**)) in rice samples after being cooked with tap water and Milli-Q water. The median concentrations and mean concentrations are represented by straight horizontal lines and small circles, respectively, and the boxes are defined by the 25th and 75th percentiles.

In China, tap water and purified water are most frequently utilized during the rice cooking process [38]. We thus used tap water and Milli-Q water to obtain the influence of different types of water through rice cooking. The rice sample containing high concentrations of m-NEOs (Σm-NEOs = 2.38 ng/g) was selected for the estimation of the RRs of m-NEOs in this study. Specifically, the rice sample was equally divided and cooked with tap water and Milli-Q water (seven samples in each group), respectively. The RRs of ∑p-NEOs and ∑m-NEOs (Figure 3) were also calculated (Table S2). The difference in RRs of ∑p-NEO observed between the samples cooked with tap (59.10 ± 19.02%) and Milli-Q water (59.82 ± 12.74%) was inconspicuous. However, the RR of the ∑m-NEOs were 90.86 ± 9.35% when using tap water, whereas that using Milli-Q decreased to 72.21 ± 26.57%, though the difference was insignificant. The results suggest that m-NEOs could be effectively eliminated through the cooking process, especially when cooking with tap water which contains various impurities, including chlorine. This result also reveals the reason for the low DRs and concentrations of m-NEOs in cooked rice.

A few studies indicated that the stabilities of m-NEOs are inferior to that of p-NEOs. Research on coffee beans revealed that the roasting process reduces the DIN concentrations by 62.2–100%, while the metabolites, including DIN-U, are almost completely eliminated after the heat treatment process [39]. Another composting experiment indicated that IMI is continuously transformed during the composting process, and olefin-IMI was observed in the compost after five days, but no olefin-IMI remained on the tenth day [40]. The main degradation pathways of IMI are photolysis, hydrolysis, and biodegradation, and IMI degradation is generally active in alkaline conditions [40]. Compared with IMI, olefin-IMI has an unstable double bond, which significantly contributes to water loss during hydrolysis, while IMI mainly experiences bond homolysis via NO_2_• loss [41]. Therefore, m-NEOs are more unstable during thermal, chemical, or biological processes than p-NEOs, and nearly 100% of m-NEOs might be removed, which is consistent with our results. In addition, some other pesticides could also be degraded and eliminated during the heat treatment progress, such as dichlorvos, diazinon [42], endosulfan, and hexachlorobenzene [43]. Many researchers, including us, suppose that heat treatment could reduce the potential exposure to pesticides through the pathway of diet.

### 2.3. Fates of NEOs in Water through Heat Treatment Process

We also calculated the RR value of the NEOs in drinking water to obtain their alteration in the heat treatment process. The concentrations of ACE (RR: 11%), CLO (RR: 4%), and THD (RR: 4%) decreased slightly, but the reductions in THM (RR: 40%) and DIN (RR: 37%) were significant (*p* < 0.05, *t*-test, Table S3 and Figure 2). The reductions in p-NEOs in the boiled tap water sample might directly reveal the stabilities of different p-NEOs, but the studies on NEOs reductions in tap water are still limited. A previous study suggested that the hydrolysis rate of THM in water is much higher than those of IMI, ACE, and CLO at 21.5 °C [44], and DIN could also experience hydrolysis through different pathways in the water phase [45]. Therefore, THM and DIN might present fewer stabilities in tap water than ACE, IMI, CLO, and THD, especially during the heating process, which might promote hydrolysis and pyrolysis.

**Figure 4:**
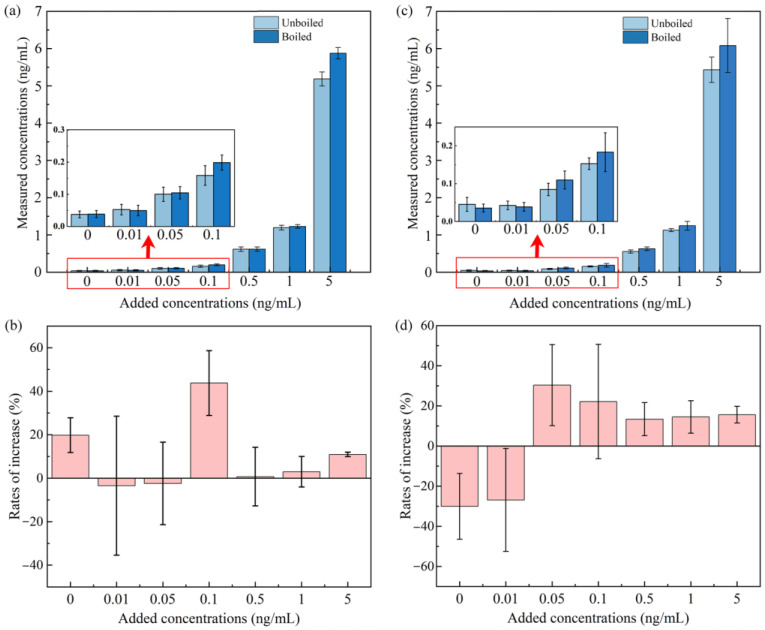
Median concentrations of IMI measured before and after the boiling process in tap water (plot (**a**)) and Milli-Q water (plot (**c**)), and the mean rates of increase in tap water (plot (**b**)) and Milli-Q water (plot (**d**)).

Interestingly, we observed a significant (*p* < 0.05, *t*-test) increase in IMI (approximately 13%, Table S3) in the tap water samples through the heat treatment process. Moreover, the data on the IMI concentrations were separately analyzed in the tap water samples collected from six districts in Guangzhou. The median rates of increase (IRs) range from 2% to 34% (Figure 2). Thus, a series of spiked experiments were performed to determine the potential sources of IMI. Different concentrations of the standard were added to the tap water samples. The concentrations measured before and after the boiling process are shown in Figure 4. The median concentrations shifted higher in most of the groups of tap water (Figure 4), and the background values of the concentrations were determined in the non-spiked group (0.037 and 0.038 ng/mL were detected in unboiled and boiled samples, respectively). Obviously, IMI concentration changes were irregular in tap water. The research on the precursors of IMI is still limited to the industrial synthesis process. For example, nitropolychlorobutadienes, N-(6-chloropyridin-3-yl)methylethylenediamine, and 1,1-bis(methylsulfanyl)-2-nitroethene are supposed to be important precursors of IMI in the industrial synthesis process [46]. However, the evidence of precursor transformation in water and the precursors source are still lacking, and we mainly attribute the contamination of precursors to the standard applied in this study, which might carry a trace of precursors through the industrial synthesis process.

The IMI concentration increases should present a positive correlation with the standard-spiked concentrations of IMI when the spiked experiment was repeated in the Milli-Q water samples if the inference mentioned above is reasonable. Subsequently, a set of Milli-Q water samples were added at different concentrations of the IMI standard, and IMI increases were also observed in the samples with a spike value above 0.05 ng/mL (Figure 4). Obviously, the IMI concentrations increased by −0.004, 0.03, 0.03, 0.07, 0.12, and 0.65 ng/mL in the spiked groups of 0.01, 0.05, 0.1, 0.5,1, and 5 ng/mL, respectively. The mean increase rates of these groups are −26.90%, 30.33%, 22.12%, 13.38%, 14.48%, and 15.59%, respectively (Figure 4). The background values of the IMI concentrations were determined in the non-spiked groups (0.045 and 0.036 ng/mL detected in unboiled and boiled samples, respectively), while the mean reduction rate of this group was 30%, and the result of this group excludes the possibility of the pollution caused by nonstandard operation or container contamination. These results in the Milli-Q water samples prove that IMI indeed increased during the heating process. Furthermore, we performed linear fitting for the spiked and increased concentrations, and the two variables are significantly proportional (r^2^ > 0.99, *p* < 0.0001). We could almost confirm that the concentration increases of IMI were caused by the IMI native standard, which might contain unidentified precursor substances. Therefore, these precursors might be generated similarly during the NEO insecticides production process and discharged into the surface along with rainfall [47], then entering urban water.

In addition, the increases were irregular and unstable in the tap water samples due to the more complex chemical composition of tap water than that of Milli-Q water. Specifically, the effect of the free chlorine in tap water is significant, the half-life of IMI in water with low chlorine concentration is ~70 days, and IMI would reduce by order of magnitude within 150 h [19]. However, the residual organic matter and HCO3- in tap water might obstruct the hydrolysis of IMI [48], and these results suggest that the fate of IMI in tap water is more complex.

As described above, IMI degraded in the rice samples after the cooking process, and degradation might have also occurred in the boiling process of all the water samples. The terminal concentration changes can be calculated according to the difference between the transformed concentrations of the precursors and those of IMI. According to existing data, a concentration-based IMI activity in water was assumed: (1) Instead of the transformation of precursors, the degradation of IMI dominated the heat treatment process of the water samples when the concentrations of IMI were comparatively low (≤0.01 ng/mL, Figure 4). (2) Given that the concentrations of IMI were comparatively high (≥0.05 ng/mL, Figure 4), the transformation of the precursors obscured the effect of IMI degradation during the heat treatment process of the water samples. To the author’s knowledge, this is the first study that revealed the increase in IMI in water samples after the heat treatment process.

## 3. Materials and Methods

### 3.1. Chemicals and Reagents

The native standards of six p-NEOs (>97% purity), namely, ACE, IMI, CLO, THD, THM, and DIN, were purchased from Sigma-Aldrich Chemical (St. Louis, MO, USA), while three m-NEOs (>98% purity), namely, N-dm-ACE, olefin-IMI and DIN-U, were purchased from Dr. Ehrenstorfer (Augsburg, Germany). Internal standards (>97% purity) ACE-d3, IMI-d4, CLO-d3, THD-d4, THM-d3, and DIN-d3 were also obtained from Sigma-Aldrich Chemical (St. Louis, MO, USA). Acetonitrile (ACN), methanol (MeOH, HPLC grade), formic acid (>95% purity), primary secondary amine (PSA), and graphitized carbon (GCB) were purchased from CNW Technologies Inc. (Shanghai, China). Milli-Q water was obtained from the Milli-Q system (Millipore; Billerica, MA, USA).

### 3.2. Sample Collection

A total of 35 rice samples were obtained from Qinyuan City (n = 20) and Guangzhou City (n = 15) in Guangdong Province, China. The rice samples (500 g each) were randomly collected from local markets and canteens, shipped to the laboratory, sealed, and stored at room temperature (25 °C) until analysis.

A total of 30 tap water samples (n = 30) were obtained from six districts (i.e., Haizhu district, HZ; Yuexiu district, YX; Conghua district, CH; Tianhe district, TH; Huadu district, HD; Liwan district, LW) of Guangzhou (n = 5 in each district). The tap water samples (500 mL each) were randomly collected from canteens and family residences, shipped to the laboratory, and stored in polypropylene (PP) tubes at −20 °C until analysis.

### 3.3. Rice Sample Preparation

The extraction process was implemented according to the studies of Botías et al. [49] and Iwafune et al. [50].

Uncooked rice samples: Two grams of polished rice sample were transferred into a 15-mL PP tube. A mixed internal standard solution (20 μL, 1 μg/mL) and 5 mL of ACN were added to the sample, and the mixture was vortexed for 2 min. Then, 1 g of NaCl was added, and the mixture was vortexed for another 1 min. Subsequently, the mixture was centrifuged for 5 min (5000 rpm). The supernatant was transferred into a new 15-mL PP tube (containing 20 mg PSA and 20 mg GCB), vortexed for 30 s, then centrifuged for 1 min (5000 rpm). The supernatant was transferred to another 15-mL PP tube, concentrated to dryness under a gentle stream of nitrogen, redissolved in 0.5 mL of ACN, and then filtered with a 0.22 μm filter (CNW Technologies Inc., Shanghai, China). The final extract was transferred into a 2-mL vial for HPLC-MS/MS analysis.

Cooked rice samples: 200 g of rice sample was heated (100 °C) with 240 mL of tap water (obtained from the laboratory) in a pre-cleaned cooker for 20 min. In addition, the special cooker applied in this study could ensure that ~95% of the condensed water was returned to the sample. Subsequently, the cooked rice sample was lyophilized for 36 h, and 2 g of the lyophilized sample was polished into powder. The following extraction operations were performed on the polished powder as described above.

### 3.4. Water Sample Preparation

The tap water samples were prepared in duplicate following the studies of He et al. [14] and Mahai et al. [51].

Tap water samples (NEOs-examination experiments): An internal standard solution (50 μL, 0.1 ng/μL) was added to 500 mL of the water sample, and the mixture was filtered with a 0.45-μm filter (Shanghai XINYA Purification Equipment Co., Ltd., Shanghai, China). The water sample was loaded onto an HLB cartridge (500 mg, 6 mL, CNW Technologies Inc., Shanghai, China), which was pre-conditioned with 5.0 mL of MeOH and 5.0 mL of Milli-Q water and infiltrated at 3.0 mL/min. Then, the cartridge was rinsed with 5.0 mL of Milli-Q water, vacuumed, and dried for 5 min. The target chemicals were eluted with 5.0 mL of MeOH. The extract was concentrated to dryness under a gentle stream of nitrogen, redissolved in 0.2 mL of ACN, and filtered with a 0.22-μm filter. The final extract was transferred into a 2-mL vial for HPLC-MS/MS analysis. For the heat treatment, the water examples were heated to 100 ℃ (1 atm) and stopped heating immediately, then cooled down to room temperature before the extraction process, and the following extraction operations were as described above.

Tap water and Milli-Q water samples (spiked experiments): Tap water and Milli-Q water samples were extracted and not subjected to the concentration process. In summary, a native standard solution of IMI (150 μL; 0.01, 0.05, 0.1, 0.5, 1, and 5 ng/μL, separately) and an internal standard solution of IMI-d4 were added to 150 mil of the water sample. Then, 1 mL of water was taken out of the sample and transferred into a 2 mL vial for HPLC-MS/MS analysis. The remaining water sample was boiled and cooled down to room temperature, and 1 mL of the boiled water sample was transferred into a 2 mL vial for HPLC-MS/MS analysis.

### 3.5. Instrumental Analysis

As mentioned in our previous study [14], an Agilent 1290 series high-performance liquid chromatography (HPLC, Agilent Technologies, CA, USA) system and an Applied Biosystems SCIEX 5500 triple quadrupole mass spectrometry (MS/MS) in positive electrospray ionization mode (ESI+; Applied Biosystems, Foster City, CA, USA) were utilized to analyze the target chemicals. A Zorbax SB-C18 column (100 mm × 2.1 mm, 3.5 μm; Agilent) was used for separation. The mobile phase consisted of ACN (solvent A) and water (0.1% formic acid, solvent B). Multiple reaction monitoring (MRM) mode was used for the quantification of p-NEOs and m-NEOs, and the optimized MS/MS parameters are listed in Table S4.

### 3.6. Quality Assurance and Quality Control

Six procedural blanks and six instrumental blanks (i.e., ACN/water injection) were analyzed to eliminate the contamination arising from the reagents, the glassware, and the instrument. The contamination levels of NEOs in the blanks (<1.0 ng/L for DIN and <0.10 ng/L for other NEOs) were less than 0.10 ng/g in the rice samples and less than 0.10 ng/L in the water samples. In order to correct the recoveries of all analytes in cooking and boiling experiments, another group of matrix-spiked samples was spiked with the analytes (10 ng/g in uncooked/cooked rice samples and 10 ng/L in unboiled/boiled tap water samples) and fortified with the internal standard solution (10 ng/g in uncooked/cooked rice samples and 10 ng/L in unboiled/boiled tap water samples), then extracted following the treatment for NEOs-examination experiments without heating process. All the recoveries were corrected by referencing the internal standard. The mean matrix-spiked recoveries (n = 3) ranged from 82 ± 14% to 91 ± 10% (rice samples) and 73 ± 18% to 95 ± 11% (water samples). The calibration curves of the target analytes were obtained over a concentration range of 0.05–50.0 ng/mL, with the regression coefficients (r^2^) above 0.99. Then, 10 ng/L of the mixed internal standard solution was injected to ensure the stability of the detector response during instrumental analysis, and the relative standard deviation was confirmed to be below 10%. The limits of quantification (LOQ), which were defined as ten times the signal-to-noise (S/N) ratio, ranged from 0.001 ng/L to 0.01 ng/L.

### 3.7. Statistical Analysis

The total concentrations of the six p-NEOs (i.e., ACE, IMI, CLO, THD, THM, and DIN) and the three m-NEOs (i.e., N-dm-ACE, olefin-IMI, and DIN-U) were denoted as Σp-NEOs and Σm-NEOs, respectively. LOQ/2 was used for the calculation of the median, the geometric mean (GM), and the arithmetic mean when the concentration was below LOQ. Data analysis was performed in SPSS Version 21.0. Datasets were tested by *t*-test (when homogeneity of variance was met) and Mann–Whitney test (when homogeneity of variance was not met). A value of *p* < 0.05 denotes statistical significance.

## 4. Conclusions

In summary, p-NEOs and m-NEOs were frequently detected in rice and water samples. The degradation of p-NEOs and m-NEOs in the rice samples during the heat treatment process could be inferred, which is more significant than that in the water samples. The types of water utilized for cooking could affect the degradation efficiency of ∑m-NEOs in rice (tap water: nearly 100%; Milli-Q water: nearly 80%). For drinking water, significant reductions in THM and DIN can be induced by the heat treatment process. The concentrations of IMI increased during the heat treatment process when the spiked concentrations exceeded 0.05 ng/mL. However, the concentrations in domestic water are very low that the increase in IMI might be negligible after the heat treatment process. In summary, human exposure to NEOs through rice and drinking water can be partially controlled by the heat treatment process.

These findings reveal that the concentrations of p-NEOs and m-NEOs could be shifted by the heat treatment process, especially in rice. Human intake can also be shifted by the process, and a part of ∑NEOs intake through rice and water can be significantly reduced. For drinking water, significant reductions in THM and DIN can be induced by the heat treatment process. Although the concentrations of IMI increased in some spiked groups, the concentrations in domestic water are very low that the increase in IMI might be negligible after the heat treatment process. In summary, human exposure to NEOs through rice and drinking water can be partially controlled by the heat treatment process.

## Figures and Tables

**Table 1:** Concentrations of NEOs in uncooked and cooked rice (ng/g) were collected from Qingyuan and Guangzhou.

		p-NEOs						Σp-NEOs ^a^	m-NEOs			Σm-NEOs ^b^	
		ACE	IMI	CLO	THD	THM	DIN		DIN-U	N-dm-ACE	Olefin-IMI		ΣNEOs ^c^
	LOQ	0.001	0.01	0.01	0.003	0.01	0.01		0.01	0.01	0.01		
Uncooked rice	DR (%) ^d^	91	80	71	71	89	69	97	54	46	46	74	97
	median	10.4	10.5	11.8	9.79	11.5	10.5	66.8	0.06	<LOQ	<LOQ	0.51	71.4
	mean	12.3	13.8	16.8	11.8	14.2	13.5	82.3	0.40	8.33	10.33	19.1	101
	GM ^e^	1.55	1.85	1.58	0.88	2.07	1.24	12.4	0.06	0.17	0.19	0.82	19.2
	min	<LOQ ^f^	<LOQ	<LOQ	<LOQ	<LOQ	<LOQ	0.02	<LOQ	<LOQ	<LOQ	0.02	0.04
	max	74.5	92.5	114	71.2	86.9	92.9	532	2.62	38.0	59.9	91.4	532
Cooked rice	DR (%)	89	71	66	66	74	57	97	60	40	34	80	97
	median	6.38	6.39	8.58	5.77	6.24	7.41	41.4	0.09	<LOQ	<LOQ	0.36	44.1
	mean	7.03	7.31	9.45	6.56	7.45	7.97	45.8	0.40	5.62	6.88	12.9	58.7
	GM	0.81	0.64	0.68	0.29	0.61	0.45	5.58	0.06	0.08	0.08	0.64	8.42
	min	<LOQ	<LOQ	<LOQ	<LOQ	<LOQ	<LOQ	0.02	<LOQ	<LOQ	<LOQ	0.02	0.04
	max	25.0	20.3	32.2	22.0	28.1	23.3	141	4.14	33.7	47.1	77.8	180

^a^ The sum concentrations of six p-NEOs. ^b^ The sum concentrations of three m-NEOs. ^c^ The sum concentrations of nine NEOs (p-NEOs + m-NEOs). ^d^ Detection rate. ^e^ Geometric mean. ^f^ Below the limits of quantification (LOQ).

## Data Availability

The data presented in this study are available in the article and the Supplementary Materials.

## Supplementary Materials

The following supporting information can be downloaded at: https://www.mdpi.com/article/10.3390/molecules28104194/s1, A supplementary document containing four tables is provided. The Median concentrations of six p-NEOs in uncooked rice and the steam collected during the heat treatment process are listed in Table S1. The RRs of p-NEOs and m-NEOs in rice samples after being cooked with tap water and Milli-Q water are listed in Table S2. The median concentrations of p-NEOs and m-NEOs in tap water samples and their RRs before and after being boiling process are listed in Table S3. The detailed information on optimized MS/MS parameters for all analyzed NEOs is listed in Table S4.
