# Supplementary material for: Changing on the Concentrations of Neonicotinoids in Rice and Drinking Water through Heat Treatment Process
Source: Molecules. 2023 May 19;28(10):4194. doi: 10.3390/molecules28104194 (PMC10223057; doi:10.3390/molecules28104194)
Supplement: Supplementary file 1 [file molecules-28-04194-s001.zip › molecules-2385868-supplementary.pdf]

# Supplementary Materials

## Changing on the Concentrations of Neonicotinoids in Rice and Drinking Water through Heat Treatment Process

Ziyang Wei[1], Bo Zhang[1], Xu Li[1], Yanxia Gao[1], Yuan He[1], Jingchuan Xue[2], Tao Zhang[1*]

[1]*School of Environmental Science and Engineering, Sun Yat-Sen University, Guangzhou 510275, China*

[2]*Key Laboratory for City Cluster Environmental Safety and Green Development of the Ministry of Education, School of Ecology, Environment and Resources, Guangdong University of Technology, Guangzhou, 510006, China*

**Corresponding authors:**

*Tao Zhang

School of Environmental Science and Engineering, Sun Yat-Sen University

135 Xingang West Street, Guangzhou, 510275, China

Tel: 86-22-84113454

E-mail: zhangt47@mail.sysu.edu.cn

**Table S1.** Median concentrations of six p-NEOs in uncooked rice, and the steam collected during heat treatment process

| | **Uncooked rice (ng/g)** | **Steam (ng/L)** |
|---|---|---|
| **ACE** | 5.68 | < LOQ |
| **IMI** | 6.19 | < LOQ |
| **CLO** | 5.92 | 0.002 |
| **THD** | 5.37 | < LOQ |
| **THM** | 5.94 | 0.012 |
| **DIN** | 6.28 | 0.003 |
| **DIN-U** | 0.63 | < LOQ |
| **N-dm-ACE** | 0.03 | < LOQ |
| **olefin-IMI** | 1.81 | < LOQ |

**Table S2.** Reduction rates (RRs) of p-NEOs and m-NEOs in rice samples after being cooked with tap water and Milli-Q water

| | | ∑ p-NEOs | ∑ m-NEOs |
|---|---|---|---|
| **Cooked with tap water** | **tap-1** | 57.88% | 100.00% |
| | **tap-2** | 68.68% | 97.14% |
| | **tap-3** | 73.71% | 98.16% |
| | **tap-4** | 62.22% | 97.42% |
| | **tap-5** | 66.23% | 78.53% |
| | **tap-6** | 67.53% | 84.57% |
| | **tap-7** | 17.47% | 80.19% |
| **Cooked with Milli-Q water** | **Milli-Q-1** | 37.14% | 46.23% |
| | **Milli-Q-2** | 75.00% | 90.80% |
| | **Milli-Q-3** | 67.52% | 94.28% |
| | **Milli-Q-4** | 50.63% | 75.99% |
| | **Milli-Q-5** | 64.54% | 76.38% |
| | **Milli-Q-6** | 67.15% | 95.80% |
| | **Milli-Q-7** | 56.75% | 25.96% |

**Table S3.** Median concentrations (ng/L) of p-NEOs and m-NEOs in tap water samples and their reduction rates (RRs) before and after being boiling process

| | **Unboiled** | **Boiled** | **RR** |
|---|---|---|---|
| **ACE** | 7.28 | 6.50 | 11 % |
| **IMI** | 26.0 | 29.5 | -13 % |
| **CLO** | 10.2 | 9.76 | 4 % |
| **THD** | 0.23 | 0.22 | 4 % |
| **THM** | 41.3 | 24.8 | 40 % |
| **DIN** | 12.3 | 7.76 | 37 % |
| **DIN-U** | < LOQ | < LOQ | 0 % |
| **N-dm-ACE** | < LOQ | < LOQ | 0 % |
| **olefin-IMI** | < LOQ | < LOQ | 0 % |

**Table S4.** Optimized MS/MS parameters for all analyzed neonicotinoids (NEOs)

| NEOs | Parent ion (m/z) | Product ion (m/z) | Cone voltage (V) | Collision energy (eV) | Internal standards |
|---|---|---|---|---|---|
| **ACE** | 223 | 126 (56) [a] | 140 | 17 (13) [b] | ACE-$d_3$ |
| **IMI** | 256 | 209 (175) [a] | 80 | 10 (18) [b] | IMI-$d_4$ |
| **CLO** | 250 | 169 (132) [a] | 80 | 12 (6) [b] | CLO-$d_3$ |
| **THD** | 253 | 126 (186) [a] | 140 | 16 (10) [b] | THD-$d_4$ |
| **THM** | 292 | 211 (181) [a] | 80 | 6 (22) [b] | THM-$d_3$ |
| **DIN** | 203 | 129 (87) [a] | 80 | 8 (14) [b] | DIN-$d_3$ |
| **DIN-U** | 159 | 85 (67) [a] | 80 | 14 (16) [b] | DIN-$d_3$ |
| **N-dm-ACE** | 209 | 126 (90) [a] | 70 | 26 (12) [b] | ACE-$d_3$ |
| **olefin-IMI** | 254 | 152 (171) [a] | 70 | 18 (12) [b] | IMI-$d_4$ |

[a] The numbers outside the brackets indicate quantification ions, while those in the brackets indicate identification ions. [b] The numbers outside the brackets indicate the collision energy of quantification ions, while those in the brackets indicate that of identification ions.
